# First Description of Sarcoptic Mange in a Free-Ranging European Wildcat (*Felis silvestris silvestris*) from Spain

**DOI:** 10.3390/ani11092494

**Published:** 2021-08-25

**Authors:** Fernando Nájera, Elena Crespo, Amalia García-Talens, Rebeca Grande-Gómez, Francisco Javier Herrera-Sánchez, Michaela Gentil, Carmen Cortés-García, Elisabeth Müller, Rafael Calero-Bernal, Luis Revuelta

**Affiliations:** 1 Department of Animal Physiology, Faculty of Veterinary Medicine, Complutense University of Madrid, 28040 Madrid, Spain; lrevuelt@vet.ucm.es; 2 Asistencia Técnica de la Dirección General del Medio Natural y Desarrollo Sostenible de la Junta de Comunidades de Castilla-La Mancha, Plaza del Cardenal Siliceo s/n, 45071 Toledo, Spain; ecrespo@jccm.es (E.C.); amgata@hotmail.com (A.G.-T.); rgrandegomez@gmail.com (R.G.-G.); javher80@gmail.com (F.J.H.-S.); 3 “El Chaparrillo” Wildlife Rehabilitation Center, Ctra de Porzuna s/n, 13071 Ciudad Real, Spain; 4 “La Alfranca” Wildlife Rehabilitation Center Finca de la Alfranca s/n, Pastriz, 50195 Zaragoza, Spain; 5 Laboklin GmbH & Co. KG, Steubenstrasse 4, 97688 Bad Kissingen, Germany; gentil@laboklin.com (M.G.); cortes@laboklin.com (C.C.-G.); mueller@laboklin.com (E.M.); 6 SALUVET, Animal Health Department, Faculty of Veterinary Medicine, Complutense University of Madrid, Avenida Puerta de Hierro s/n, 28040 Madrid, Spain

**Keywords:** European wildcat, *Felis silvestris*, mange, *Sarcoptes scabiei*, Spain

## Abstract

**Simple Summary:**

Sarcoptic mange caused by the mite *Sarcoptes scabiei* is a worldwide-distributed skin infestation with a wide range of hosts, among them several species within the Felidae family. *Sarcoptes scabiei* was diagnosed in a dead adult female European wildcat (*Felis silvestris silvestris*) from Spain. This is the first description of *Sarcoptes scabiei* in a European wildcat. Since this is a species of conservation concern due to its critical demography in the southernmost population of the Iberian Peninsula, the impacts of infectious diseases, including sarcoptic mange, should be considered during disease surveillance programs of the species’ populations.

**Abstract:**

Sarcoptic mange caused by the mite *Sarcoptes scabiei* is a worldwide-distributed skin infestation with a wide range of hosts, among them several species within the Felidae family. *Sarcoptes scabiei* was diagnosed in a dead adult female European wildcat (*Felis silvestris silvestris*) from Spain, based on histological evaluation of skin biopsies and identification of the arthropod from skin scrapings and molecular methods. This is the first description of *Sarcoptes scabiei* in a European wildcat. Due to its critical demography in the southernmost population of the Iberian Peninsula, the impacts of infectious diseases, including sarcoptic mange, as a new potential threat should be considered during disease surveillance programs of the species’ populations.

## 1. Introduction

Sarcoptic mange caused by arthropods of the genus *Sarcoptes* is a highly contagious skin disease that has been observed to affect nearly 150 species of mammals, leading to acute or chronic forms depending on factors such as host immunity and mite lineage [1,2]. Its relevance relies on an economic, zoonotic and ecological basis [3,4,5]. Additionally, sarcoptic mange could represent a potential threat to be considered within the Carnivore guild, which is already of conservation concern, since 14% (15/108) of the species under suboptimal conservation status within the order Carnivora are considered susceptible to this disease [2]. Sarcoptic mange has been described in several species of wild felids, including the African lion (*Panthera leo*), the cheetah (*Acinonyx jubatus*), the serval (*Felis serval*), the leopard (*Panthera pardus*), the Eurasian lynx (*Lynx lynx*) and the Iberian lynx (*Lynx pardinus*) [6,7].

The European wildcat (*Felis silvestris silvestris*) remains a widely distributed felid species in Europe, although it is assumed to be an endangered taxa in most of the countries in which it lives [8]. The demographic situation of the southernmost population of the species in the Iberian Peninsula is somewhat critical, primarily due to low density and habitat fragmentation [9]. Under these circumstances, the species may benefit from health surveillance to detect disease threats for the populations. The present study reports the first observation of a case of sarcoptic mange in a European wildcat.

## 2. Materials and Methods

On 1 November 2020, during a routine field surveillance performed under the Iberian lynx conservation and reintroduction program in Castilla-La Mancha (South Central Spain), an adult (>2-year-old) female wildcat was found dead in a private estate that consisted of Mediterranean scrubland in Las Virtudes (Ciudad Real province, 859857X 340946Y). The dead wildcat was found in right lateral recumbency with no signs of struggle and no injuries. The animal was brought to El Chaparrillo Wildlife Rehabilitation Center (Ciudad Real), kept in refrigeration and necropsied within 12 h after harvesting.

On physical examination, we discarded a putative individual based on coat patterns from dorsal, lateral, ventral, head and tail, showing a pelage characteristic typical of true wildcats [10,11,12]. The carcass suffered from an initial stage of decay and was calculated between 3–4 days of postmortem interval. It weighed 2.4 kg and it was given a 1/5 body condition score. Skin lesions were clearly visible on the head, and both tarsus and skin scrapings were taken from both pinnae and tarsus for microscopic examination and direct detection under light microscopy (x40–100). Two 5 × 5 cm patches of skin were submitted to the laboratory to perform molecular testing for *S. scabiei* via Taqman real-time PCR (qPCR), targeting the internal transcribed spacer (ITS)-2 region (primer Sarco S: 5′-GCT AAA GAA TCC AAG TGC CA-3′, Sarco R: 5′-TCT TTT CCT CCG CTT ATT TAT ATG-3′, probe Sarco TM: 5′-6FAM-CGG GTA TTC TCG CTT GAT CTG AGG TC-BBQ-3′). Prior to nucleic acid extraction, skin samples were incubated in a lysis buffer (containing proteinase K) in “MagNA Lyser Green Bead” tubes (Roche Diagnostics, Mannheim, Germany), which contain ceramic beads and crush the tissue through mechanical disruption (MagNA Lyser, Roche Diagnostics, Mannheim, Germany). Afterwards, automated total nucleic acid extraction was carried out using a commercially available kit (“MagNA Pure 96 DNA and Viral NA Small Volume Kit”, Roche Diagnostics, Mannheim, Germany). Each PCR run included a negative and a positive control, as well as an extraction control in each sample, to check for nucleic acid extraction and PCR inhibition.

Additionally, portions of skin samples were fixed in 10% buffered formalin and submitted to histopathological analyses by standard hematoxylin and eosin staining.

In addition, aiming for ancillary analyses, several tissue samples from mesenteric ganglia, spleen, bone marrow, clot, kidney and lung were taken to perform PCR analysis and/or culture for selected pathogens; blood (1 mL) was also taken to perform feline leukemia virus p27 antigen and feline immunodeficiency antibodies point-of-care enzyme-linked immunosorbent assays, and liver for anticoagulant rodenticide analysis (Table 1).

## 3. Results

**Figure 1:**
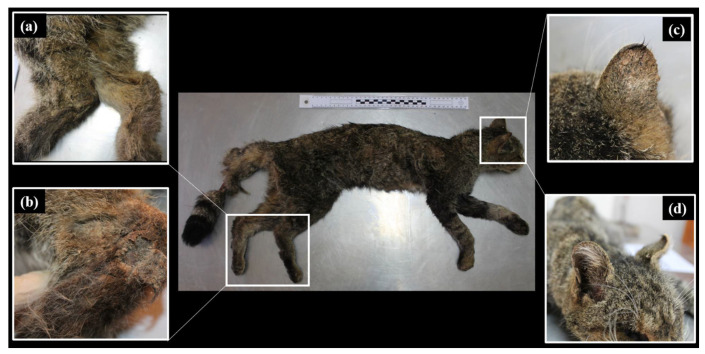
Physical aspect of the dead female European wildcat and most relevant macroscopic alterations reported during the necropsy. (**a**) Thickening of both tarsal joints. (**b**) Detail of hyperkeratosis of the left proximal tarsus. (**c**) Thickening and crusting in right pinnae. (**d**) Loss of hair density in the rostral plane.

On gross examination, skin revealed thickening of the edge of both pinnae, slight crusting of both pinnae, loss of hair density in nasal and rostral planes and thickening and crusting of the skin of both tibia–tarsal joints. The only alopecic area found in the carcass corresponded to the central area of the tail, but it was considered a post-mortem sign (Figure 1). On examination of the subcutaneous tissue, congestion was observed in the rostral plane, both tarsus and proximal area of the tail. In addition, severe muscle mass loss and minimal body fatty stores were observed. The lungs and liver presented several 1 × 1 mm white foci scattered in their serosa.

**Figure 2:**
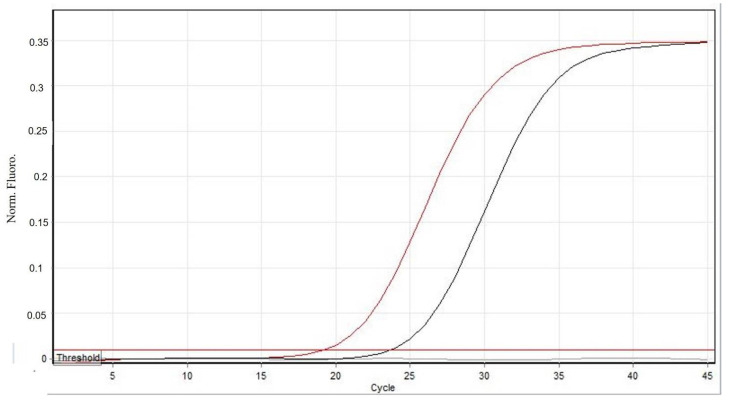
Result of the Taqman real-time PCR for *Sarcoptes scabei* var. *canis*; black = positive control, grey = negative control, red = skin sample from the European wildcat.

By means of light microscopy examination, mites detected in the skin scrapes were identified as the adult stage of *Sarcoptes scabiei* according to morphological identification keys [21]. Nevertheless, skin patches submitted to the laboratory were processed to confirm mite identification by molecular methods; an early amplification was shown by the qPCR (Ct value: 19.3) indicating a high parasite load in the samples (Figure 2).

**Figure 3:**
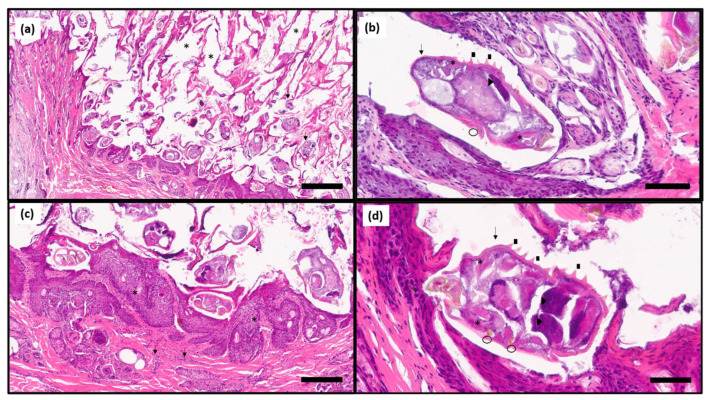
(**a**): The stratum corneum is expanded by an up to 4 mm thick layer of parakeratotic hyperkeratosis embedding numerous tunnels (three of them are marked with asterisks) that contain many cross and tangential sections of arthropods (two of them are marked with black arrows) Barr = 600 µm. (**b**,**d**): Arthropods (black arrows) are approximately 250 × 150 µm in size, have a thick chitinous exoskeleton, dorsal spines (black squares), a hemocoel, striated muscle (asterisks), jointed appendages (circles) and digestive and reproductive organs (arrow heads). Numerous eggs with a tan shell obliterated with basophilic globular material are visible. Barr = 60 and 80 µm, respectively. (**c**): Associated with the parasites, the superficial dermis is diffusely expanded by moderate numbers of perivascular to interstitial neutrophils, macrophages, eosinophils, fewer lymphocytes and plasma cells (arrow). The epidermis is moderately hyperplastic (asterisk). Barr = 200 µm. H&E stain.

Histological examination revealed moderate hyperplastic epidermis, stratum corneum expanded by a thick layer of parakeratotic hyperkeratosis embedding numerous mite tunnels and superficial dermis diffusely expanded by moderate numbers of perivascular to interstitial neutrophils, macrophages, eosinophils, fewer lymphocytes and plasma cells (Figure 3). The high number of mites observed per section is in agreement with the low Ct value observed in the qPCR.

Results from the ancillary tests performed in the European wildcat are expressed in Table 1.

## 4. Discussion

According to arthropod identification, clinical signs, histological examination and parasite DNA amplification, a case of sarcoptic mange by *Sarcoptes scabiei* was confirmed for the first time in a European wildcat. In this case, we used reliable diagnostic tools to identify the ectoparasite. As a limitation, carcass preservation prevented enough blood extraction to obtain serum to carry out an indirect ELISA, used for the immunodiagnosis of *S. scabiei* in various animal species [7,22,23,24,25,26].

Although specific molecular diagnosis was performed for *S. scabiei*, its lineage identity discrimination was not achieved. Host specificity could be relevant to predict sarcoptic mange virulence in novel hosts and explore cross-species transmission dynamics [2]. The area where the carcass was retrieved represents a fair example of an anthropogenic landscape, a suitable scenario for cross-species disease transmission. *Sarcoptes scabiei* cross-species transmission has been reported for 38% (*n* = 56) of the known host species [2]. In Spain, red foxes are endemic hosts for sarcoptic mange [27], and in this region wildcats are sympatric to this canid, making fox–wildcat transmission the most plausible route of infestation. However, the role of domestic dogs, cats or even rabbits (a keystone prey species in the Mediterranean ecosystem) as a mange source for this individual cannot be ruled out [28,29]. Within a cross-species transmission network, domestic species present higher connectivity and may have a key role in *S. scabiei* transmission pathways to wildlife hosts [2].

As for transmission dynamics, direct or indirect exposure may be considered in our case. Direct contact via predator–prey species, intraguild predation or carrion consumption have been proposed as non-social transmission mechanisms in solitary species [30,31,32,33,34,35]. Indirect transmission (i.e., environmental transmission) may be enabled by adequate microclimate of resting areas, dens and burrows used by foxes or badgers, both sympatric to European wildcats [34,36,37].

The development of clinical signs in our case may be due to a reduction in resistance to infestation, and environmental stress of this individual, but we cannot rule out the role of a concomitant disease, body condition and/or stressing environmental conditions on the development of immune response, as observed in other wildlife species [2,4]. We cannot determine the relevance of the anticoagulant (brodifacoum) exposure in this wildcat but we hypothesized that, even at the low levels detected (0.04 ppm), it might play a role due to the sublethal effects of these compounds. Evidence of both inflammatory response and immune suppression associated with anticoagulant rodenticide exposure could influence susceptibility to opportunistic infections as observed in Southern California bobcats (*Lynx rufus*) [38]. This finding also raises concern in relation to the potential exposure to rodenticides of another sympatric endangered felid in this region, the Iberian lynx.

We could not define the ultimate cause of death in this case, since some ancillary tests are lacking in this study necessary to aid in the diagnosis (e.g., bacterial culture/histopathology of liver and lung). Due to its poor body condition, and gross necropsy findings, sepsis could have played a role as it has been frequently found to be related to sarcoptic-mange-affected wildlife, as a consequence of immunosuppression [39,40,41,42]. A secondary bacterial infection could have developed by immunosuppression due to sarcoptic mange, or due to the immune suppression derived from anticoagulant rodenticide exposure [38].

## 5. Conclusions

The European wildcat population across its range faces several threats to its long-term survival due to habitat loss, roadkill, hybridization with its domestic counterpart and disease transmission [43,44,45]. In the Mediterranean Iberian Peninsula, other threats include the loss of its main prey, the European wild rabbit, *Oryctolagus cuniculus,* and the low density and fragmentation of its populations [9,46,47]. Under this scenario of silent extinction, understanding the impact of diseases such as sarcoptic mange is paramount, since the introduction of parasites to naïve host populations can result in drastic population declines and localized extinctions [48,49]. Further research is warranted to monitor this disease in European wildcats in order to recognize the potential risk that sarcoptic mange could represent for the conservation of the species.

## Figures and Tables

**Table 1:** Additional diagnostic tests performed in the female European wildcat (*Felis silvestris silvestris*) found dead in Ciudad Real province, Spain, in November 2020.

Analyte ^1^	Tissue Sample	Result	Test	Laboratory and/or Reference
FPV	Mesenteric ganglia	Negative	PCR	Laboklin [13]
FCoV	Intestinal scraping samples	Negative	PCR	Laboklin [14]
FHV-1	Clot, spleen	Negative	PCR	Laboklin
FCV	Clot	Negative	PCR	Laboklin [15]
FeLV provirus	Clot, mesenteric ganglia, bone marrow	Negative	PCR	Laboklin [16]
FIV	Clot	Negative	PCR	Laboklin [17]
P27 Ag FeLV	Blood	Negative	ELISA	IDEXX SNAP^®^ Combo Plus, IDEXX Laboratories, ME, USA
FIV Ab	Blood	Negative	ELISA	IDEXX SNAP^®^ Combo Plus, IDEXX Laboratories, ME, USA
CDV	Clot	Negative	PCR	Laboklin [18]
*Leishmania* spp.	Spleen	Negative	PCR	Laboklin [19]
*Mycobacterium* spp.	Lung	Negative	Culture	Regional Agricultural Laboratory-LARAGA
*Leptospira* spp.	Kidney	Negative	PCR	Laboklin [20]
Anticoagulant rodenticide	Liver	Positive	HPLC-MS/MS	Regional Agricultural Laboratory-LARAGA

^1^ FPV = feline panleukopenia virus, FCoV = feline coronavirus, FHV-1 = feline herpesvirus 1, FCV = feline calicivirus, FeLV = feline leukemia virus, FIV = feline immunodeficiency virus, p27 Ag FeLV = feline leukemia virus p27 antigen, FIV Ab = feline immunodeficiency virus antibodies, CDV = canine distemper virus, PCR = polymerase chain reaction, ELISA = enzyme-linked immunosorbent assay, HPLC-MS/MS = high-performance liquid chromatography with tandem mass spectrometric.

## Data Availability

Data supporting reported results are available upon request to authors.
